# Influence Mechanism of Surfactants on Wettability of Coal with Different Metamorphic Degrees Based on Infrared Spectrum Experiments

**DOI:** 10.1021/acsomega.1c02954

**Published:** 2021-08-18

**Authors:** Yingying Hu, Qingtao Zhang, Gang Zhou, Haiyang Wang, Yanlong Bai, Yejiao Liu

**Affiliations:** † Binzhou University, Binzhou 256600, China; ‡ College of Safety and Environmental Engineering, Shandong University of Science and Technology, Qingdao 266590, China; § State Key Laboratory of Mining Disaster Prevention and Control Co-founded by Shandong Province and the Ministry of Science and Technology, Shandong University of Science and Technology, Qingdao 266590, China; ∥ School of Civil Engineering, Chongqing Jiaotong University, Chongqing 400074, China; ⊥ Chengjiao Coal Mine of Yongmei Company of Henan Energy and Chemical Industry Group, Yong Cheng 476600, China; # Institute of Mining Research, Inner Mongolia University of Science and Technology, Baotou 014010, China

## Abstract

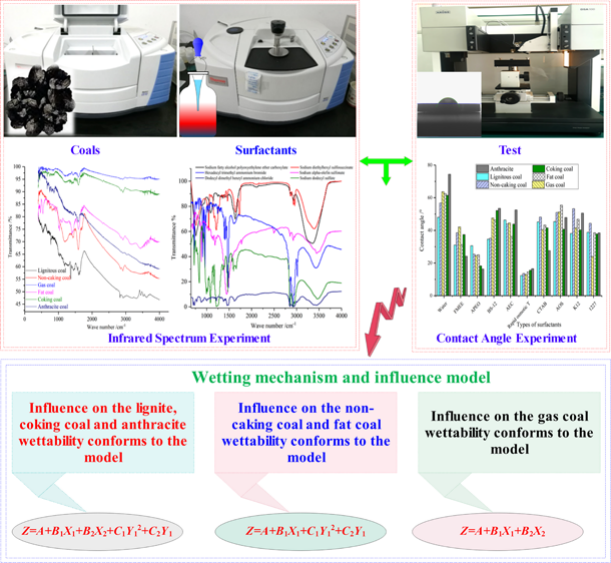

Based on experiments, a numerical analysis is used to quantitatively explore the influence of coal and surfactant microstructures on wettability. First, based on an infrared spectrum experiment, the distribution of oxygen-containing functional groups, aromatic hydrocarbons, and aliphatic hydrocarbons of coal and surfactants was obtained. Second, the wettability relationship between coal and different surfactants was determined by optical titration, and the coal dust wettability and surfaces were optimized. The key factors of the active agent wetting ability affecting lignite wetting mainly depend on the carbonyl, ether, and carboxyl groups in the surfactant. The factors affecting non-stick coal and gas coal wetting mainly depend on the ether group and aromatic amine in the surfactant. The factors affecting fat coal wetting mainly depend on the ether group and hydroxyl group in the surfactant. Finally, the factors affecting coking coal and anthracite wetting mainly depend on the surfactant ether group, aliphatic amine, and aromatic amine. Then, combining the structural parameters with the coal wetting results, the quantitative mathematical relationship between coal dust wettability, the important influencing factors of the surfactant, and the wettability index was established. Finally, a perfect and reasonable wettability evaluation model between coal and the surfactants was established. The relative activity of methyl ether and aromatic ether is greater than that of methyl ether, and the influence on the lignite, coking coal, and anthracite wettability conforms to the model . The influence on the non-caking coal and fat coal wettability conforms to the model , while the influence on the gas coal wettability conforms to the model *Z* = *A* + *B*_1_*X*_1_ + *B*_2_*X*_2_. In general, this study provides scientific guidance for the compounding of high-efficiency and environmentally protective composite dust suppressors to realize clean mine production.

## 1 Introduction

Coal dust is an increasingly serious problem in underground coal mines. Traditional mine dust prevention measures have limited effectiveness and have not achieved the desired results. In recent years, with the rise of chemical dust suppression, mine dust has been effectively controlled.^1−3^ Since its first use, chemical dust suppression has been applied to a variety of dust control sites in a relatively new and effective manner.^4,5^ In the 1920s, sulfonated hydrocarbon was used by British scholars in a mine, which contributed to dust management at that time.^6^ In the 1930s, the US Bureau of Mines began applying wetting agents to the prevention and control of coal mine dust. However, since 1974, the US Bureau of Mines^7^ has carried out measurements of the practical application of wetting agents on a long-walled working face, but the results are not satisfactory. In some studies, it has been found that even if the same dust suppressant is applied to different working surfaces, the results are different.^8,9^

Glanviller and Haley^10^ used the Walker test to study various factors affecting the wetting rate of coal dust. The results show that the wetting rate is mainly related to the temperature, the particle size composition of coal dust, and the surfactant used. The type and characteristics are related. Kilau and Pahlman^11^ found that coal wettability is highly correlated with the type and content of minerals contained in coal. When a suitable sodium or potassium salt is added to the anionic surfactant, the affinity of the surfactant to wet the coal is greatly enhanced, and the coal wettability is remarkably improved. Singh^12^ used surfactants for the adsorption kinetics and adsorption isotherms of coal dust surfaces to demonstrate the interaction between coal and surfactants at a microscopic level, revealing the reasons for changing wettability. Elkin and Istomin^13^ studied coal particle wetting resulting from pulverized coal lumps to substantially change the structural and surface properties of the coal, leading to a substantial change in its mechanical, physical, and chemical properties. Akti and Unal^14^ used Zonguldak bituminous coal and Sivas-Divrigi Ulucayir (SDU) lignite as adsorbents for the adsorption of nonionic Igepal CA-630. Kinetic and equilibrium studies were carried out at initial concentrations between 3 and 50 ppm for 24 h.

In China, the main measure for controlling dust in mines in the past 20 years has been the use of dust suppressants. In 1992, Ren et al.^15^ studied the effects of wettability and adhesion of liquid spreading on the dustproof effect; Du et al. of Beijing University of Science and Technology^16^ developed a dust suppressant that suppresses dust from open coal yards based on the adhesion of liquids. Xie and Li^17^ and Jin et al.^18^ mixed starch with sodium dodecylbenzene sulfonate and glycerol to obtain a relatively efficient and environmentally friendly dust suppressant. Yang et al.^19^ used fatty alcohol polyoxyethylene ether, polyvinyl alcohol, and film-forming auxiliaries as raw materials to develop a new type of polymer dust suppressant with wind and rain resistance. After spraying on the coal surface, a continuous dense polymer microfilm can form on the coal pile in a short time, which has a good bonding effect and good dust, wind, and rain resistance. Wang et al.^20^ investigated the surface tension and contact angle of surfactants under different magnetization conditions, and the results showed that the magnetization of dust suppressants not only maintains their good wettability but also decreases their required concentration by half. Guo et al.^21^ believes that the decrease in hydrophilicity of the lignite surface can be achieved by treating with surfactant. Zhou et al.^22−41^ used sodium alginate as the base and conducted chemical modification through the grafting copolymerization technique to prepare an agglomeration-based dust suppressant with decent liquidity and wettability. Moreover, the molecular chain of lignin is modified by a chemical modification method to prepare a coal mine dust suppression product that has a series of functions, such as dust reduction, covering, and dust adhesion. To effectively improve the wetting ability of water used for coal dust suppression, surfactant-magnetized water was proposed by Qin et al.^42^ because of the synergy between magnetization and surfactants. The wetting features of this product were systematically studied under various preparation parameters. Yao et al.^43^ and Xu et al.^44^ investigated quantitative data of carbon- and oxygen-containing groups of lignite, gas coal, and anthracite through nuclear magnetic resonance (NMR) and X-ray photoelectron spectroscopy (XPS) experiments. Liu et al.^45^ analyzed the adsorption of hexadecyltrimethylammonium bromide (abbreviation: CTAB) to organic content and mineral matter and discussed the relationship between the distribution characteristics of different functional groups and the inhibiting efficiency in hydrophilicity. Liu et al.^46,47^ believed that the adsorption of surfactants onto lignite surfaces may result in wettability changes and slow the reabsorption of moisture onto dried lignite. Although the above research has made some progress, on one hand, the wetting mechanism of coal dust and surfactant has not been clearly stated. On the other hand, the coupling relationship between coal dust and the surfactant and the key factors affecting wettability have not been examined. However, due to the complexity of its wetting mechanism, research on coal dust wetting is not deep and thorough, which makes it difficult to make a breakthrough in dust reduction.

Based on Fourier transform infrared spectroscopy experiments, this study selected coal samples from different coal ranks in six mines in China and nine different types of surfactants for infrared spectroscopy experiments to develop coal dust surface functional groups and different dust suppressants. The functional groups and contents of coal and surfactants with different degrees of metamorphism in typical mining areas in China have been obtained. After that, through the statistical analysis of the test data by the numerical analysis and calculation tool, the important indexes affecting the wettability of coal dust are extracted, and the coupling relationship between the multiple parameters of coal dust wettability and physical and chemical characteristics is fitted to determine the influence law of the microphysical and chemical characteristics of coal dust on its wettability. Then, through the determination and analysis of the coal and surfactant wettability, the influencing factors of the coal and surfactant wettability and its evaluation model are obtained. While avoiding the waste of resources caused by the combination of dust suppressants, this study also provides scientific guidance for the combination of dust suppressants. The results have important theoretical and practical significance for guiding the research and development of mine dust suppressants.

## 2 Experimental Materials and Methodology

### 2.1 Preparation of Coal Samples

To make the coal sample representative on one hand and ensure its richness on the other hand, we selected six coal samples with different coal ranks as experimental coal samples. Table 1 shows the details of the coal types and sampling mines.

**Table 1:** Coal Samples

coal type	sample point	station
lignitous coal	4301 working face of Beizao coal	Longkou, Shandong
non-caking coal	311,306 working face of Bayan gaole coal	Ordos, Inner Mongolia
gas coal	1206 working face of Jinrun coal	Tongchuan, Shanxi
fat coal	1301N working face of Xinjulong coal	Heze, Shandong
coking coal	2560 working face of Xinzhi coal	Huozhou, Shanxi
anthracite	8124 working face of Yangquan coal	Yangquan, Shanxi

Onsite research and study of multiple mines at home and abroad was completed, combined with the current situation of surfactants commonly used in the coal industry. Through the comprehensive analysis of various factors such as the price, cost, effect, and ecological characteristics of different surfactants, the final results point to nine kinds of surfactants. Table 2 shows the surfactant type details.

**Table 2:** Types of Surfactant

number	name of surfactant	abbreviation	type	chemical molecular formula
S1	fatty methyl ester ethoxylate	FMEE	non-ionic surfactant	C_18_H_36_CO(OCH_2_CH_2_)_7_OCH_3_
S2	alkylphenol ethoxylates	APEO		CH_3_(CH_2_)*_x_*C_6_H_4_(OC_2_H_4_)*_y_*OH
S3	amphiprotic surfactant BS-12	BS-12		C_16_H_33_NO_2_
S4	sodium fatty alcohol polyoxyethylene ether carboxylate	AEC	ionic surfactant	RO(CH_2_CH_2_O)_10_CH_2_COONa (R = C_12–14_)
S5	sodium diethylhexyl sulfosuccinate	Rapid osmotic *T*		C_20_H_36_O_7_SNa
S6	hexadecyltrimethylammonium bromide	CTAB		C_19_H_42_BrN
S7	sodium alpha-olefin sulfonate	AOS		RCH=CH(CH_2_)_n_—SO_3_Na (n = C_14–16_ or C_14–18_)
S8	sodium dodecyl sulfate	K12		C_12_H_25_SO_4_Na
S9	dodecyl dimethyl benzyl ammonium chloride	1227		C_21_H_38_NCl

### 2.2 Infrared Spectroscopy Experiment

**Figure 1:**
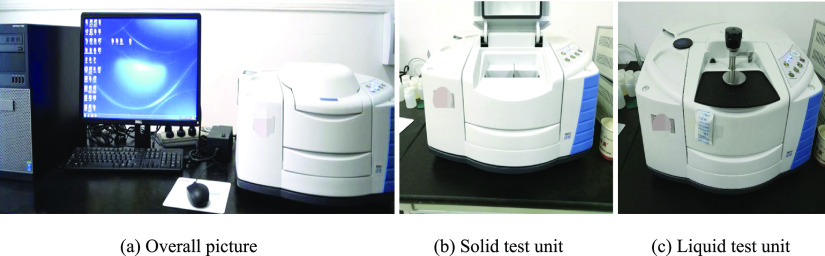
Nicolet iS10 Fourier infrared spectrometer. (Photograph courtesy of Qingtao Zhang. Copyright 2021. Images are free domain.)

Samples used in this article were tested using a Nicolet iS10 Fourier infrared spectrometer (shown in Figure 1). When testing solid coal samples, first, the solid test unit of the instrument was connected. Then, the solid sample was pulverized together with potassium bromide and pressed into a sheet in a dedicated mold for measurement. Its operation method is as follows: after thoroughly grinding 100 mg of potassium bromide crystal with an agate mortar, 1 mg of coal was added to the sample to be tested, and the sample was mixed and ground until uniform. The particle size was smaller than the length of the detected light wave (approximately 2 μm or less); a circular paper ring was placed on a metal mold with a polished surface; and the ground powder was moved into the ring with a spatula. Another mold was covered and put into the tableting machine for tableting; a transparent sheet was made of 0.1–1.0 mm thickness; the sheet was fixed with the sample holder; and the sample was placed in the infrared spectrometer for testing. Cumulative scanning is performed 32 times to obtain the infrared spectrum. When testing a liquid sample with a Nicolet S10 Fourier infrared spectrometer, the liquid test unit on the instrument should be connected. Since the selected surfactant samples were chemically pure or the individual samples became the analytical reagent, it was not necessary to purify the test samples, and the liquid test with the Nicolet iS10 Fourier infrared spectrometer could be directly used to test the liquid sample.

### 2.3 Experiment on Wettability Measurement of the Coal and Surfactant

**Figure 2:**
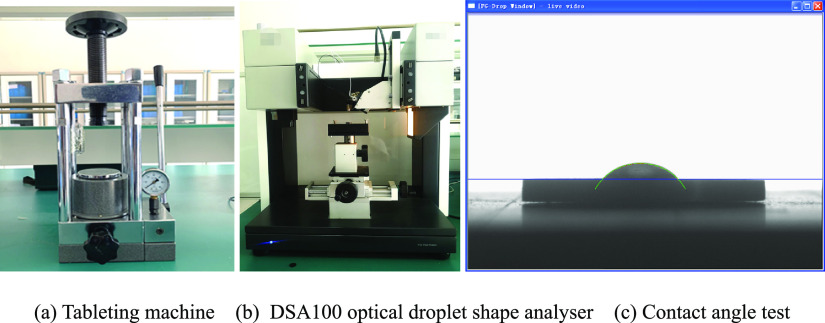
Determination of wettability between coal and surfactant. (Photograph courtesy of Qingtao Zhang. Copyright 2021. Images are free domain.)

In this paper, the DSA100 optical droplet morphology analysis system was used to determine the dynamic contact angle of surfactant and coal, as shown in Figure 2. The steps are as follows: using a tableting machine, 2.3 g of coal powder with a particle size of approximately 2 was pressed into a cylindrical sample with a diameter of 13 mm and thickness of 1 mm under a pressure of 20 MPa. The sample to be tested was fixed on the test platform, and the syringe was installed. The position of the needle and the shape of the droplet were controlled by the control panel; the baseline and contact angle were measured; and the final result was read by the instrument.

## 3 Experimental Results and Analysis

### 3.1 Fourier Infrared Spectroscopy Analysis of Various Coals

**Figure 3:**
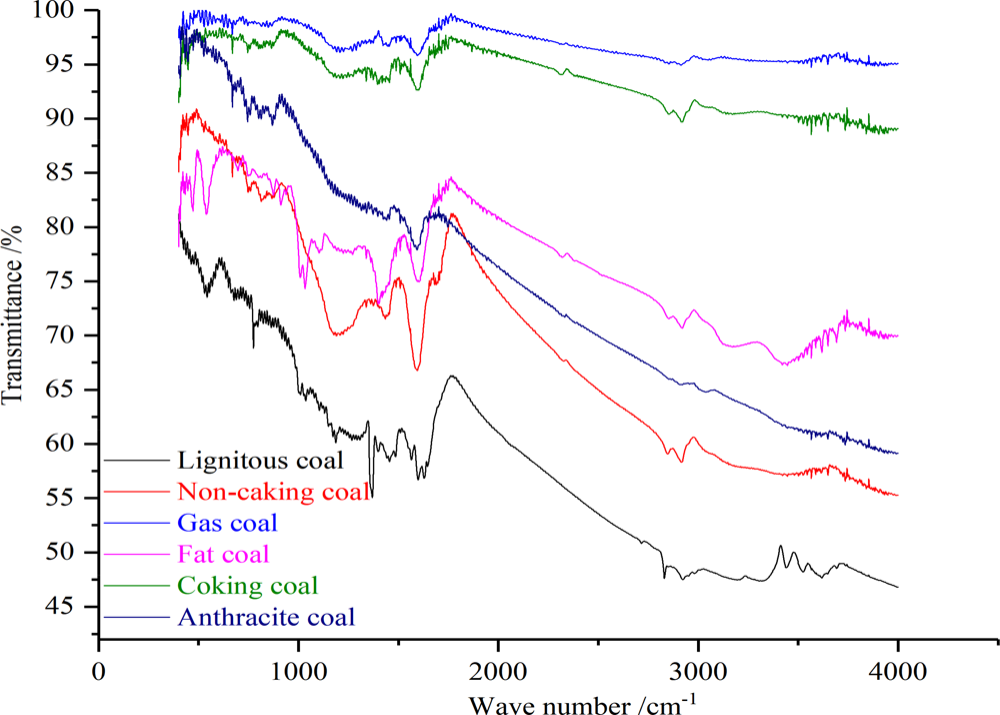
Infrared spectra of different metamorphic coal types.

Through infrared spectra examination of the coal samples, it was found that with the evolution of coal ranks, the infrared spectrum of coal also underwent regular changes, which indicates that the functional groups in coal change with the evolution of coal rank as well as the differences between coal types. In this paper, Fourier infrared spectra experiments were carried out on six coal samples of different coal ranks to obtain the infrared spectra, shown in Figure 3.

Figure 3 shows that the vibration peak of the oxygen-containing functional group in lignite is more obvious, and in the lignite, the total amount of aromatic hydrocarbons and aliphatic hydrocarbons is less. In non-caking coal, the substitution peak of the methyl group on the benzene ring is obvious, and the vibration absorption peak of the carbonyl and benzene ring is obvious at 1584 cm^–1^. The vibration absorption peaks of the carbon oxygen single bond at 1205–1265 cm^–1^, ether group at 1100 cm^–1^, and hydroxyl group at 3500–3650 cm^–1^ are obvious. In fat coal, the aromatic ring at 985–1025 cm^–1^ and hydroxyl group at 3500–3650 cm^–1^ are obvious, and there are double bond absorption vibration bands at approximately 1610 and 1697 cm^–1^. The obvious absorption of the aromatic ring in coking coal is 3035–3238 cm^–1^, which indicates that there are more aromatic hydrocarbons in coking coal. The absorption peak of oxygen-containing functional groups in anthracite coal is not obvious, but there are a large number of vibration absorption peaks between 1376–1716 and 2880–3000 cm^–1^, indicating that the content of aromatic hydrocarbons and aliphatic hydrocarbons is rich.

### Fourier Infrared Spectroscopy Analysis of Different Surfactants

**Figure 4:**
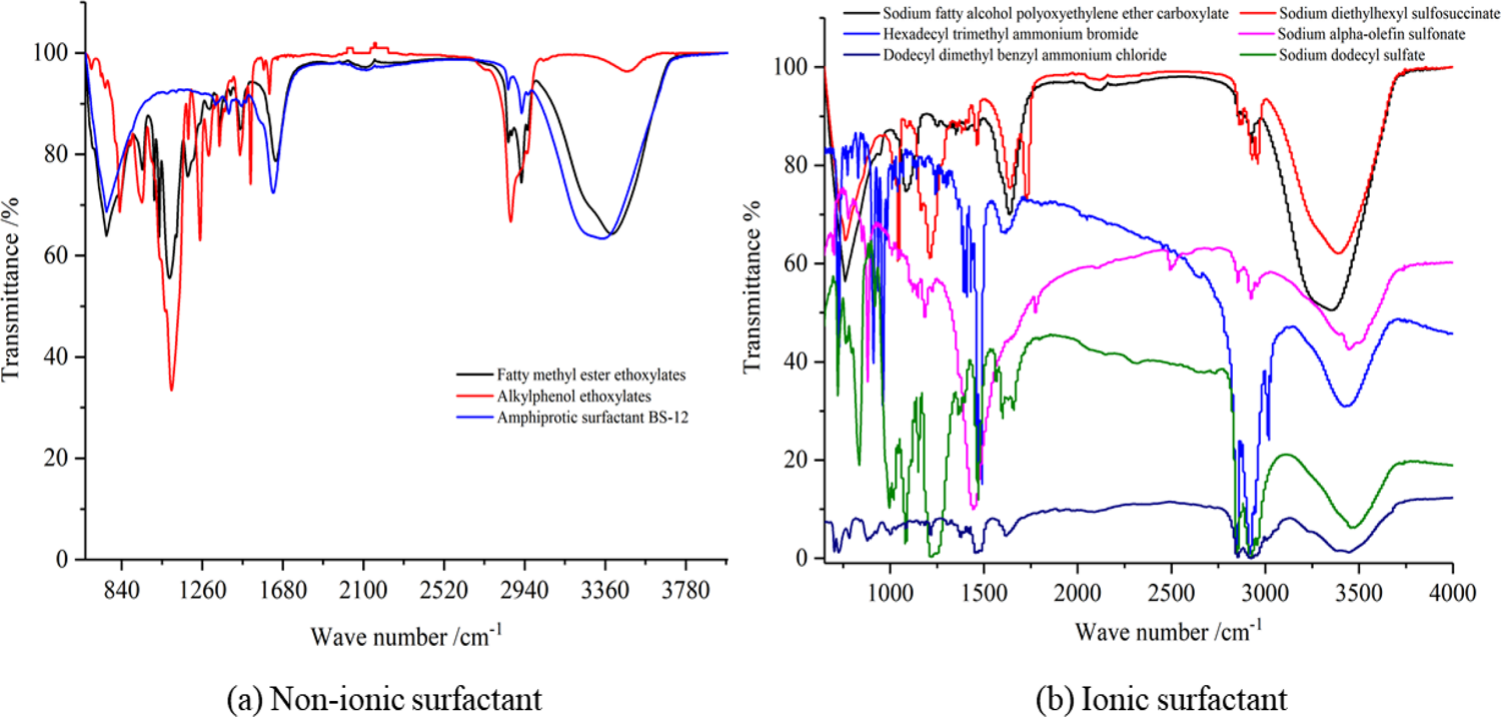
Infrared spectra of surfactants.

In this paper, nine kinds of surfactants were tested by infrared spectroscopy, as shown in Figure 4.

Figure 4 shows that FMEE contains many oxygen-containing functional groups, among which the hydroxyl group content is the most abundant. BS-12 contains a large amount of oxygen-containing functional groups, among which the hydroxyl group is most abundant. Rapid osmotic *T* contains many oxygen-containing functional groups, among which the hydroxyl group content is the most abundant. The content of aromatic hydrocarbons of AOS is rich, and the content of oxygen-containing functional groups is small, but the amount of hydroxyl groups is the most. AEC also contains many oxygen-containing functional groups, among which the content of ether groups is the most abundant. CTAB is rich in aliphatic hydrocarbons, while the content of oxygen-containing functional groups is less, but the content of hydroxyl groups is the most abundant. K12 contains more aromatic amines and oxygen-containing functional groups, among which CO– and hydroxyl groups are the most abundant.

### 3.2 Distribution of Functional Surfactant Groups

**Figure 5:**
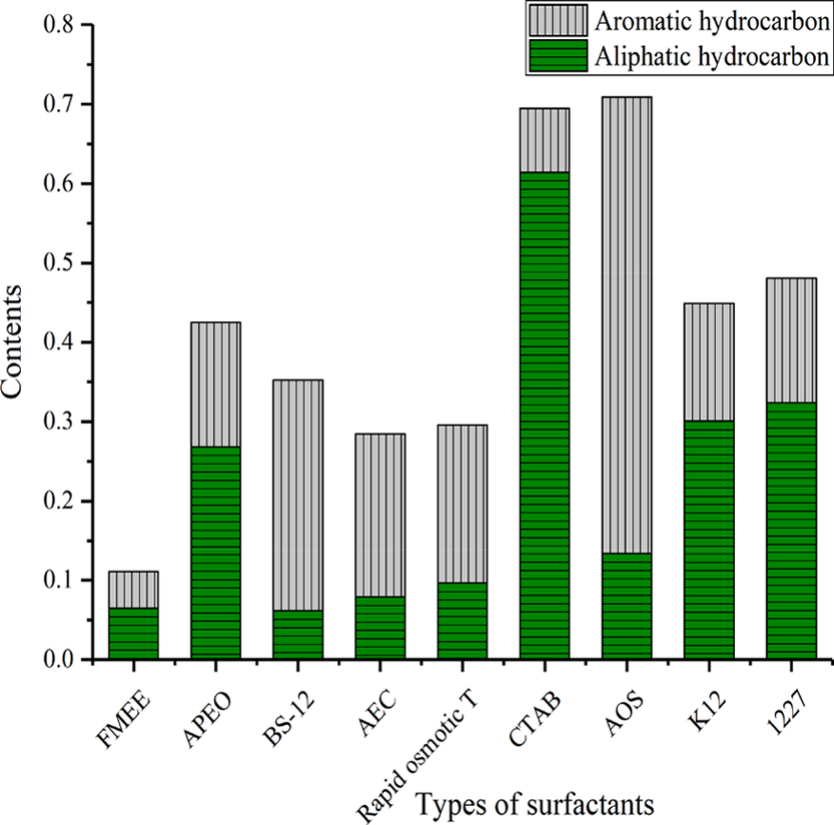
Content of aromatic hydrocarbons and aliphatic hydrocarbons.

**Figure 6:**
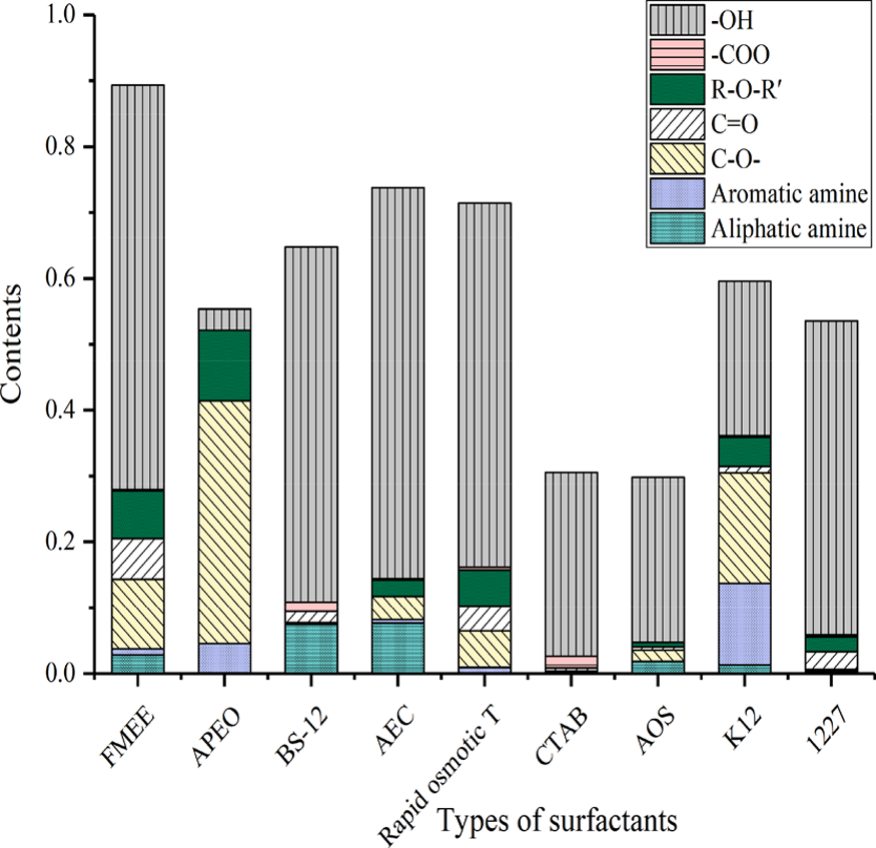
Oxygen functional group and amine functional group content.

After the peak-fitting of the selected nine surfactants is carried out, the specific functional groups contained in the different surfactants can be obtained. The specific content of the functional groups of the different surfactants is shown in Figures 5 and 6.

Figures 5 and 6show that in FMEE, the content of hydroxyl groups is the majority, accounting for 62%, followed by C–O– and the carbonyl and ether groups; the content of aromatic hydrocarbons and aliphatic hydrocarbons is relatively small. The most typical component of APEO is C–O–, accounting for approximately 37% of the total, followed by aliphatic hydrocarbons and aromatic hydrocarbons, and small amounts of ether linkages and aromatic amines. The hydroxyl content of BS-12 accounts for approximately 54% of the total followed by the aromatic hydrocarbon content of approximately 30% of the total; the total amount of fatty amines accounts for approximately 7.5% of the total, and the total amount of aliphatic hydrocarbons accounts for approximately 6% of the total. The hydroxyl group of AEC is approximately 59% of the total functional groups followed by aromatic hydrocarbons accounting for 20.5% of the total, aliphatic hydrocarbons accounting for 8% of the total, fatty amines accounting for 7.6% of the total, and carbon–oxygen single bonds accounting for 3.5% of the total. Rapid osmotic *T* has a rich distribution of oxygen-containing energy-carrying groups, in which hydroxyl groups account for 55% of the total; CO– accounts for 5.5% of the total; carbonyl accounts for 3.7% of the total; ether bonds account for 4.5% of the total; fatty amines account for 20% of the total; and aliphatic hydrocarbons account for 9.6% of the total. The main component of CTAB is aliphatic hydrocarbon, accounting for 61% of the total, while the aromatic hydrocarbon content is less, only 8% of the total. The distribution of oxygen-containing groups is relatively simple, showing only the absorption peaks of hydroxyl and carboxyl groups, among which hydroxyl groups account for 28% of the total, and carboxyl groups account for 1.8% of the total. In AOS, the content of aromatic hydrocarbons accounts for 57.5% of the total; aliphatic hydrocarbons account for approximately 13.3% of the total; and hydroxyl groups account for 25% of the total. In K12, aromatic hydrocarbons and aliphatic hydrocarbons account for 30 and 15% of the total, respectively. In the K12 oxygen-containing group, the hydroxyl group content is 23.5%, and the CO– content is approximately 17%. Notably, K12 contains a certain amount of aromatic amine, accounting for approximately 12.4% of the total. In 1227, the content of aromatic hydrocarbons is 15.7%, the content of aliphatic hydrocarbons is 32.4%, and the distribution of oxygen-containing groups is relatively simple, mainly hydroxyl groups. The content of hydroxyl groups accounts for 48% of the total, and the concentration of carbonyl groups accounts for 2.7% of the total.

The microphysical structure of different surfactants has different distributions. This provides a good basis and guiding choice for the construction of coal wetting and evaluation models for different coal ranks.

### 3.3 Test Results of Coal Dust and Surfactant Wettability

In the experiment, the wettability was measured by using a surfactant solution of 0.06%^1^ (this concentration is proposed on the basis of relevant scholars’ research, reaching the critical micelle concentration (CMC), which will not be discussed in detail in this paper) concentration. At the same time, to make the results more accurate, three sets of data were made, and the average value was taken as the experimental result, as shown in Table 3.

**Table 3:** Test Results of Wettability

coal surfactants	lignitous coal	non-caking coal	gas coal	fat coal	coking coal	anthracite
water	47.98	56.74	63.67	62.54	61.46	74.35
FMEE	31.03	38.54	42.14	31.22	37.4	24.18
APEO	30.62	25.41	24.53	25.03	18.24	16.35
BS-12	34.57	35.13	47.55	46.27	52.15	53.47
AEC	46.34	43.89	44.55	36.17	43.82	52.51
rapid osmotic *T*	12.35	13.55	12.98	14.58	15.62	16.54
CTAB	45.19	48.08	40.46	43.25	41.53	27.59
AOS	45.38	50.98	51.17	55.43	40.69	47.86
K12	38.12	53.26	41.46	46.81	40.3	50.56
1227	38.84	44.3	24.01	38.46	37.79	38.42

**Figure 7:**
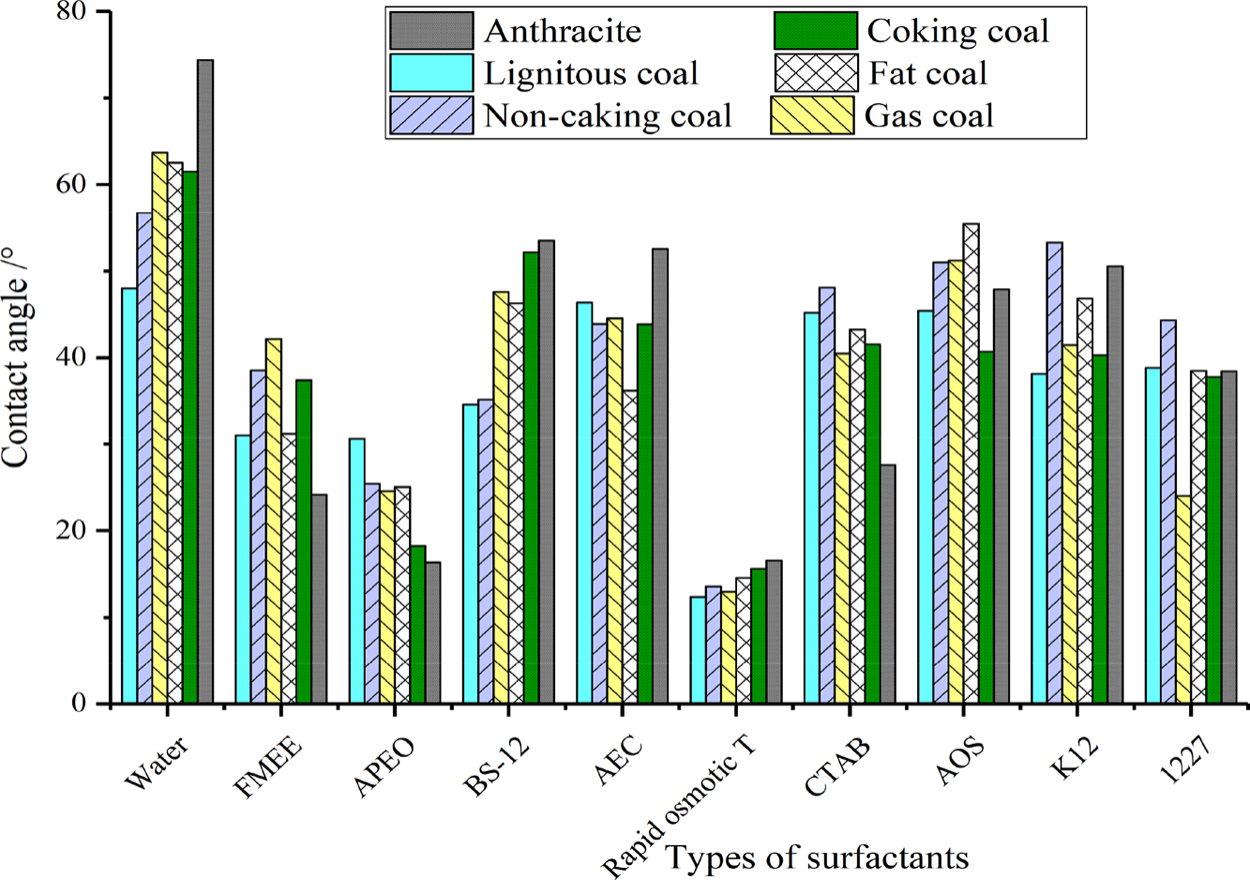
Evaluation of wettability between coal and various surfactants.

The wettability relationship between coal and each surfactant is shown in Figure 7.

Figure 7 shows that the wettability exhibited by each surfactant for different coal types is greatly different. In the water of the control experiment, the contact angle of coal and water gradually increases with the deepening of the degree of coal evolution before the surfactant is added, which indicates that as the degree of coal evolution increases, the affinity of coal to water gradually decreases.

For FMEE, as the degree of coal evolution deepens, the affinity of coal and surfactant first decreases and then rises. For APEO, as the degree of coal evolution increases, the affinity of the coal before the surfactant increases. For BS-12, the wetting behavior exhibited by BS-12 is similar to that of water, and as the degree of coal evolution increases, the affinity decreases. For AEC, it can be seen that the contact angle between lignite and anthracite is higher, and the affinity for non-stick coal, gas coal, and coking coal is similar, but it shows strong affinity at the fat coal. Rapid osmotic *T* showed strong affinity for all coal types. As the degree of coal rank evolution increased, the affinity decreased, but it was not significant. The wetting law exhibited by CTAB is similar to that of APEO. As the degree of coal evolution increases, the affinity shows an overall increasing trend. For AOS, as the degree of coal evolution increases, the wetting performance tends to decrease first and then increase. The wettability of K12 and 1227 is not obvious. As the degree of coal evolution increases, the affinity with coal decreases first and then increases and then decreases.

Generally, the wetting effect of Rapid osmotic *T* on coal with different metamorphic degrees is obviously better than that of other surfactants, which provides strong theoretical and experimental support for the optimization of dust suppressors in the process of mine dust environment treatment. The main influencing factors of these changes are analyzed from the microperspective.

## 4 Analysis of Influencing Factors of Surfactant Wetting Coal Dust

### 4.1 Analysis of Influencing Factors of Surfactant Wettability Using Infrared Spectroscopy

Since many coal samples are selected for analysis when testing the wetting properties of surfactants, it is not possible to judge the surfactant wettability based solely on the experimental results of a group of samples. In this regard, Origin software was used to analyze the factors that affect the coal wettability of different ranks by surfactant functional group parameters. The results are described as follows:

(1) According to the correlation analysis between functional group parameters and wettability of lignite samples, it can be concluded that for lignite samples, some functional surfactant group parameters have no correlation with their wettability, but the carbonyl group, ether group, and wettability show relatively strong correlation, that is, significances (bilateral) of 0.089 and 0.087, respectively.
(2) According to the correlation analysis between functional group parameters and wettability of non-caking coal samples, it can be concluded that only the ether group and its wettability show a relatively strong correlation in infrared structural parameters of surfactants for non-caking coal samples. Although other functional groups showed a certain correlation with wettability, the significance was small, and the representation was insufficient.
(3) According to the correlation analysis of surfactant functional group parameters and wettability of gas coal samples, it can be concluded that for gas coal samples, there is a certain difference between the results of significance analysis and those of low metamorphic coal samples before. The correlation between aromatic hydrocarbons, carbon oxygen single bonds, and their wettability is obviously strengthened, and fatty amine has a strong correlation with its wettability; the significance (bilateral) is 0.084.
(4) According to the correlation analysis between functional group parameters of the surfactant and wettability of fat coal samples, it can be concluded that the ether group in the surfactant has a high correlation with its wettability, while the wettability of aromatic hydrocarbon, carbon oxygen single bond, carbonyl group, and surfactant also shows a certain correlation, but the correlation between other functional groups and wettability is relatively low.
(5) According to the correlation analysis of the functional group parameters and wettability of the surfactant in coking coal samples, it can be concluded that for coking coal samples, the ether group in the surfactant has a strong correlation with wettability, which is significantly correlated at the level of 0.05 (bilateral). At the same time, fatty amine and carbon oxygen single bonds also show a strong correlation.
(6) According to the correlation analysis of functional group parameters and wettability of anthracite samples, it can be concluded that for anthracite samples, ether groups, aliphatic amines, and wettability show a strong correlation, significantly correlated at the 0.05 level (bilateral), while aromatic hydrocarbons, carbon oxygen single bonds, and carbonyl groups also have strong correlations with wettability.

It can be seen from the above analysis that although the correlation of various functional group parameters and wettability in the surfactant obtained by infrared spectroscopy is different for different coal samples, it has certain regularity. (1) The ether group in the surfactant exhibits a strong negative correlation with the ability to wet the coal body, and the higher the content of the ether group, the lower the wetting ability of the surface active. (2) Due to the difference of the test samples, the correlation between the individual functional group parameters of the surfactant and the performance of the wetted coal body is quite different, indicating that when the surfactant wets the coal body, its wetting ability changes depending on the coal rank.

### 4.2 Construction of an Evaluation Model for Influence Factors of Surfactant Wettability

The factors affecting the coal and surfactant wettability include various aspects. Experimental tests have shown that the wettability of different surfactants shows a large difference. Most of the microstructure parameters in surfactants do not show a correlation with their wetting ability, and only a small number of structural parameters show a certain correlation with wettability. Therefore, when investigating the influencing factors between the wetting ability between the coal and surfactant, it is not easy to characterize the correlation of the wetting factors by multiple linear regression equations. Polynomial fitting^48−50^ is also required for factors that do not exhibit a linear correlation but may have an effect on wetting performance to further optimize the structural parameters that affect wetting ability.

Based on the analysis of wettability influence by polynomial fitting, it can be concluded that the data obtained by infrared spectroscopy showed a good linear correlation between carbonyl, ether group, aliphatic amine, and their wetting ability. However, aromatic amines, carboxyl groups, and hydroxyl groups have nonlinear correlation with their wettability; the other parameters have not found the exact relationship with wettability; therefore, it is omitted in the construction of the evaluation model. The specific evaluation models are as follows:

(1) Evaluation model for influencing factors of lignite sample surfactant wettability.

In the wettability test of lignite samples, the carbonyl and ether groups in the surfactant are linearly related to their wetting ability, while the content of carboxyl groups is nonlinearly related to wettability. The assignment of the wettability evaluation model variables is shown in Table 4.

**Table 4:** Description of the Evaluation Model Variables of Lignite Wettability

parameter	carbonyl	ether group	carboxyl	wettability (contact angle)
variable	*X*_1_	*X*_2_	*Y*_1_	*Z*

Based on the variables, the correlation equation of surfactant wettability (contact angle) in lignite samples was constructed:
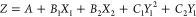
1

The fitting results are shown in Table 5.

**Table 5:** Fitting Result of the Wetting Effect Parameter of the Surfactant in the Lignite Sample

items	*A*	*B*_1_	*B*_2_	*C*_1_	*C*_2_
value	56.41743	0.06934	–2.58965	41.321	–79.37765
standard error	12.92222	0.30032	1.32793	24.4704	44.06942
statistics					
reduced chi-sqr	3.12592				
adjusted *R* square	0.45068				

Therefore, eq 1 can be expressed as follows:
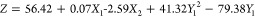
2

(2) Evaluation model for influencing factors of non-stick coal sample surfactant wettability.

In the wettability test of non-stick coal samples, the ether group, phenol or aryl ether carbon, aliphatic methyl group, and arylmethyl group in the surfactant are linearly related to their wetting ability, while the aromatic amine content is nonlinearly related to wettability. The assignment of the wettability evaluation model variables is shown in Table 6.

**Table 6:** Description of the Evaluation Model Variables of Non-stick Coal Wettability

parameter	ether group	aromatic amine	wettability (contact angle)
Variable	*X*_1_	*Y*_1_	*Z*

Based on the variables, the correlation equation of surfactant wettability (contact angle) in non-stick coal samples was constructed:
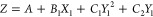
3

The fitting results are shown in Table 7.

**Table 7:** Fitting Result of the Wetting Effect Parameter of the Surfactant in the Nonstick Coal Sample

items	*A*	*B*_1_	*C*_1_	*C*_2_
value	42.53008	–3.49191	–0.19242	4.58316
standard error	8.08785	3.26809	0.94467	12.3596
statistics				
reduced chi-sqr	5.98303			
adjusted *R* square	0.13715			

Therefore, eq 3 can be expressed as follows:
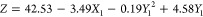
4

(3) Evaluation model for influencing factors of surfactant wettability in gas coal samples.

In the wettability test of non-stick coal samples, the ether group, phenol or aryl ether carbon, aliphatic methyl group, arylmethyl group, and fatty amine in the surfactant are linearly related to their wetting ability. The assignment of the wettability evaluation model variables is shown in Table 8.

**Table 8:** Description of the Evaluation Model Variables of Gas Coal Wettability

parameter	ether group	fatty amine	wettability (contact angle)
variable	*X*_1_	*X*_2_	*Z*

Based on the variables, the correlation equation of surfactant wettability (contact angle) in gas samples was constructed:
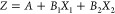
5

The fitting results are shown in Table 9.

**Table 9:** Fitting Result of the Wetting Effect Parameter of the Surfactant in the Gas Coal Sample

items	*A*	*B*_1_	*B*_2_
value	30.52522	–1.05566	2.86667
standard error	8.18956	1.28705	1.65953
statistics			
reduced Chi-Sqr	4.68242		
adjusted R square	0.31032		

Therefore, eq 5 can be expressed as follows:
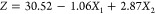
6

(4) Evaluation model for influencing factors of surfactant wettability in fat coal samples.

In the wettability test of fat coal samples, the ether group in the surfactant is linearly related to its wetting ability, and the content of hydroxyl groups is nonlinearly related to wettability. The assignment of the wettability evaluation model variables is shown in Table 10.

**Table 10:** Description of the Evaluation Model Variables of Fat Coal Wettability

parameter	ether group	hydroxyl	wettability (contact angle)
variable	*X*_1_	*Y*_1_	*Z*

Based on the variables, the correlation equation of surfactant wettability (contact angle) in the fat coal sample was constructed:
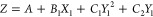
7

The fitting results are shown in Table 11.

**Table 11:** Fitting Result of the Wetting Effect Parameter of the Surfactant in the Fat Coal Sample

items	*A*	*B*_1\_	*C*_1_	*C*_2_
value	73.17062	–3.95391	0.01151	–1.14032
standard error	26.74908	1.92316	0.02081	1.49158
statistics				
reduced chi-sqr	3.2719			
adjusted *R* square	0.43375			

Therefore, eq 7 can be expressed as follows:
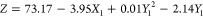
8

(5) Evaluation model for influencing factors of coking coal sample surfactant wettability.

In the wettability test of coking coal samples, the ether groups and fatty amines in the surfactant are linearly related to their wetting ability, while the content of aromatic amines is nonlinearly related to wettability. The assignment of the wettability evaluation model variables is shown in Table 12.

**Table 12:** Description of the Evaluation Model Variables of Coking Coal Wettability

parameter	ether group	fatty amine	aromatic amine	wettability (contact angle)
variable	*X*_1_	*X*_2_	*Y*_1_	*Z*

Based on the variables, the correlation equation of surfactant wettability (contact angle) in coking coal samples was constructed:
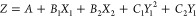
9

The fitting results are shown in Table 13.

**Table 13:** Fitting Result of the Wetting Effect Parameter of the Surfactant in the Coking Coal Sample

items	*A*	*B*_1_	*B*_2_	*C*_1_	*C*_2_
value	36.47346	–2.47304	2.2147	–0.0592	1.74462
standard error	6.96184	2.33903	1.28847	0.61154	8.04074
statistics					
reduced chi-sqr	2.57101				
adjusted *R* square	0.55102				

Therefore, eq 9 can be expressed as follows:
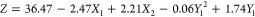
10

(6) Evaluation model for influencing factors of anthracite sample surfactant wettability.

In the wettability test of anthracite samples, the ether groups and fatty amines in the surfactant are linearly related to their wetting ability, while the content of aromatic amines is nonlinearly related to wettability. The assignment of the wettability evaluation model variables is shown in Table 14.

**Table 14:** Description of the Evaluation Model Variables of Anthracite Wettability

parameter	ether group	fatty amine	aromatic amine	wettability (contact angle)
variable	*X*_1_	*X*_2_	*Y*_1_	*Z*

Based on the variables, the correlation equation of surfactant wettability (contact angle) in anthracite samples was constructed:
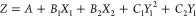
11

The fitting results are shown in Table 15.

**Table 15:** Fitting Result of the Wetting Effect Parameter of the Surfactant in the Anthracite Sample

items	*A*	*B*_1_	*B*_2_	*C*_1_	*C*_2_
value	33.57986	–2.9199	3.10679	–0.09625	3.32921
standard error	5.25235	1.69848	1.06424	0.46373	6.03902
statistics					
reduced chi-sqr	1.8227				
adjusted *R* square	0.75995				

Therefore, eq 11 can be expressed as follows:
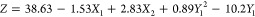
12

In this study, the microstructure of the coal and surfactant is obtained by infrared spectroscopy. The main factors affecting the wettability of the coal and surfactant are explored. Then, the micro influencing factor model of influencing factors, coal, and surfactant are established. Through the construction of a micro influence factor model of different surfactants on coal wettability, the key factors influencing coal mass with different metamorphic degrees are defined, and the correlation equation is summarized, which provides good guidance for the optimization of coal with different metamorphic degrees and the compounding of high-efficiency dust suppressors to improve the level of dust prevention and control of coal with different metamorphic degrees. At the same time, research on the dust suppressor hair has high accuracy, strong pertinence, good effect, and good science.

## 5 Conclusions

The wetting mechanism between the coal and surfactant was studied based on infrared spectroscopy experiments. The key structural parameters affecting the wetting of coal by the surfactant were clarified, and an evaluation model of the influencing factors of wettability was constructed. The main conclusions are as follows.

(1) Among the selected surfactants, rapid osmotic *T* has the best wettability, and other surfactants and different coal samples are not wetted according to the coal rank, exhibiting a decrease in wettability. In contrast, different wetting agents exhibit different wettability.
(2) Correlation analysis shows that for different coal samples, the correlation between various functional group parameters and wettability in the surfactant obtained by infrared spectroscopy is not the same but has certain regularity. (1) The ether group in the surfactant shows a strong negative correlation with the ability of wetting coal body. The higher the content of the ether group, the lower the wetting ability of the surface active. (2) The correlation between functional group parameters and wet coal performance is quite different, indicating that when the surfactant wets the coal body, its wetting ability will change according to the coal rank.
(3) According to the evaluation model of influencing factors on the wettability of the surfactant with different coal samples, it can be seen that the ether group, aliphatic amine, aromatic amine, hydroxyl group, carboxyl group, and carbonyl group in the surfactant have relatively greater influence on the wettability of coal. Among them, the ether group, carbonyl group, and carboxyl group have great influence on the wettability of lignite; the ether group and aromatic amine have great influence on the wettability of non-caking coal; the ether group and aliphatic amine have great influence on the wettability of gas coal; the ether group and hydroxyl group have great influence on the wettability of fat coal; and the ether group, aliphatic amine, and aromatic amine have great influence on the wettability of coking coal and anthracite, which can provide guidance for the compounding of high-efficiency dust suppressors.
